# microRNA analysis of gastric cancer patients from Saudi Arabian population

**DOI:** 10.1186/s12864-016-3090-7

**Published:** 2016-10-17

**Authors:** Fehmida Bibi, Muhammad I. Naseer, Sana Akhtar Alvi, Muhammad Yasir, Asif A. Jiman-Fatani, Ali Sawan, Adel M. Abuzenadah, Mohammed H. Al-Qahtani, Esam I. Azhar

**Affiliations:** 1 Special Infectious Agents Unit, King Fahd Medical Research Centre, King Abdulaziz University, Jeddah, 21589 Kingdom of Saudi Arabia; 2 Center of Excellence in Genomic Medicine Research (CEGMR), King Abdulaziz University, Jeddah, 21589 Saudi Arabia; 3 Department of Medical Laboratory Technology, Faculty of Applied Medical Sciences, King Abdulaziz University, Jeddah, Saudi Arabia; 4 Department of Medical Microbiology and Parasitology, Faculty of Medicine, King Abdulaziz University, Jeddah, Saudi Arabia; 5 Department of Anatomical Pathology, Faculty of Medicine, King Abdulaziz University, Jeddah, Saudi Arabia; 6 KACST Technology Innovation Center in Personalized Medicine, King Abdulaziz University, P.O. Box 80216, Jeddah, 21589 Kingdom of Saudi Arabia

**Keywords:** Gastric cancer, MicroRNAs, Microarray, Real time PCR, Diagnostic biomarker

## Abstract

**Background:**

The role of small non-coding microRNAs (miRNAs) in several types of cancer has been evident. However, its expression studies have never been performed in gastric cancer (GC) patients from Saudi population. First time this study was conducted to identify miRNAs that are differentially expressed in GC patients compared with normal controls.

**Methods:**

We investigated the role of miRNAs in GC patients using formalin-fixed paraffin-embedded (FFPE) tissues of 34 samples from GC patients (early stage = 7 and late-stage = 26) and 15 from normal control. We have used miRNA microarray analysis and validated the results by Real-time quantitative PCR (RT-qPCR).

**Results:**

We obtained data of 1082 expressed genes, from cancer tissues and noncancerous tissues (49 samples in total). Where 129 genes were up-regulated (*P* > 0.05) and 953 genes (*P* > 0.05) were down-regulated in 49 FFPE tissue samples. Only 33 miRNAs had significant expression in early and late-stage cancer tissues. After candidate miRNAs were selected, RT-qPCR further confirmed that four miRNAs (hsa-miR-200c-3p, hsa-miR-3613, hsa-miR-27b-3p, hsa-miR-4668-5p) were significantly aberrant in GC tissues compared to the normal gastric tissues.

**Conclusions:**

In this study we provide miRNAs profile of GC where many miRNAs showed aberrant expression from normal tissues, suggesting their involvement in the development and progression of gastric cancer. In early and late-stage miR-200c-3p showed significant down regulation as compare to control samples. Many of miRNAs reported in our study showing up-regulation are new and not reported before may be due to population difference. In conclusion, our results suggest that miR-200c-3p had potential to use as diagnostic biomarker for distinguishing GC patients from normal individuals and can be used for diagnosis of cancer at early stage.

## Background

Gastric Cancer (GC) is the second major cause for cancer-related deaths worldwide mostly prevalent in Asian countries, including Korea, Japan and China [1]. In Saudi Arabia, For all cancer types about 2.9 % is accounted for GC and it is ranked 11^th^ among both male and female population [2]. Multiple factors contribute to the progression of gastric tumors, including diet rich in salted and nitrated food, alcohol consumptions, low consumption of fruits and vegetables, use of tobacco and, especially, *Helicobacter pylori* infection [3]. Like other malignancies, both genetic and epigenetic factors are involved in the pathogenesis of gastric cancer [4]. Mortality of the disease is potentially reduces if it is diagnosed at early stages in gastric cancer [5].

Tumor markers always remain helpful at early stages in screening of high risks groups. But, the current cancer tumor markers have limited performance in detection of gastric cancer due to low sensitivity and specificity [6]. Therefore, it is required to discover some novel diagnostic biomarkers for the early detection of this malignancy. MicroRNAs (miRNAs) are small group of RNA molecules that regulate expression of different genes by binding to mRNAs. They play an important role in different cellular processes that are necessary to maintain a normal physiological condition. In cancer, particular miRNAs act as tumor supressor or oncogenic hence considered as biomarkers for early diagnosis and accurate prognosis of cancer [7]. They are considered to control a variety of functions in tumor cell including cell proliferation, migration, invasion, differentiation, apoptosis and metabolic processes [8]. Many previous studies suggest that dysregulated expression of miRNAs associated with cancers, and function as tumor inducer and suppressor and their expression may play an important role in cancer progression. The expression of miRNAs in different oncogenic pathways suggests their importance during carcinogenesis [9].

Microarray-based gene expression profiling is a potential technique to study the expression of miRNAs in GC patients [10].

Several previous studies have reported that miRNAs may be used as diagnostic biomarkers in different cancer types [11]. Fang et al. [12] has suggested some oncogenic miRNAs (miR-10b, miR-21, miR-223 and miR-338) and tumor suppressive miRNAs (miR-30a-5p, miR-126 and let-7a) as prognostic signatures in GC patients. Jiang et al. [13] has reported higher expression of miR-421 in early stage GC patients hence suggesting its role as diagnostic biomarker in GC. Using miRNA array in GC patients, abnormal expression of miRNA profiles with up and down-regulated miRNAs has been reported [14].

Although the importance of miRNAs as important prognostic factors in patients with GC is confirmed, but data on the miRNA signature of GC in the Saudi population is missing. Therefore, the present study was undertaken to detect the miRNA expression profile of GC patients and normal gastric FFPE tissue using miRNA 4.0 microarrays. In both early and late GC tumors, 33 miRNAs were found to be differentially expressed and significantly aberrants were validated using RT-qPCR.

## Methods

### Clinical samples

In this study, 34 gastric cancer tissues samples were collected from King Abdulaziz University (KAU) hospital Jeddah. Informed consent was obtained from patients undergoing a surgical procedure to remove a portion of gastric cancer. This study was approved by medical ethical committee of KAU, Jeddah Saudi Arabia (Reference#174-15). Using the tumor-node-metastasis (TNM) staging of the International Union Against Cancer (1997) [15] all the tumor samples were staged and graded according to the World Health Organization criteria [16]. From these specimens, we have collected 34 FFPE biopsy samples from gastric cancer patients and 15 FFPE gastric biopsy samples from healthy individuals along with detailed clinical history from KAU hospital (Table. 1).

**Table 1:** Clinopathological features of GC patients with normal control

Sample ID	T	N	M	Age	Sex	Tissue type
Normal						
S14-0054				50	Female	Normal
S14-0211				40	Female	Normal
S14-0267				30	Female	Normal
S14-0301				38	Female	Normal
S14-0306				31	Female	Normal
S12-0959				53	Male	Normal
S12-0851				37	Male	Normal
S12-0850				31	Female	Normal
S12-0710				47	Male	Normal
S12-0682				28	Female	Normal
S12-0669				36	Female	Normal
S12-0447				22	Male	Normal
S12-0306				26	Female	Normal
S12-0080				32	Male	Normal
S12-0049				30	Male	Normal
Early-stage						
S14.4411	1	0	0	56	Female	Cancer
S09-1886	2	0	0	78	Male	Cancer
S11-4426	2	0	0	87	Male	Cancer
S09-2859	2	0	0	52	Male	Cancer
S09-3092	2	0	0	59	Female	Cancer
S09-3841	2	0	0	72	Male	Cancer
S07-2116	2	0	0	62	Male	Cancer
Late-stage						
S13.0349-1	3	2	0	53	Male	Cancer
S13.0214-4E	3	2	0	47	Male	Cancer
S13.3130-1D	4	1	1	59	Male	Cancer
S13.3278-1D	3	2	0	70	Female	Cancer
S13.5248-2	4	3	1	55	Male	Cancer
S14.2777	2	2	0	58	Male	Cancer
S14.4235	4	3	1	18	Male	Cancer
S14.0190-1	3	2	0	77	Male	Cancer
S14.1176-1H	3	2	0	48	Male	Cancer
S09-3254	4	3	1	82	Female	Cancer
S09-3678	4	1	0	72	Male	Cancer
S09-251	3	3	0	57	Male	Cancer
S00-1751	2	2	0	55	Male	Cancer
S01-658	2	2	0	60	Male	Cancer
S02-3330	2	2	0	45	Male	Cancer
S02-3307	2	2	0	62	Male	Cancer
S02-423	3	3	1	71	Female	Cancer
S03-3123	3	3	0	54	Male	Cancer
S06-3929	3	3	0	65	Male	Cancer
S06-3390	3	3	0	27	Male	Cancer
S06-3357	3	3	0	48	Male	Cancer
S06-2460	2	1	0	81	Female	Cancer
S06-2397	2	1	0	58	Male	Cancer
S06-1949	3	2	0	43	Male	Cancer
S06-1939	3	2	0	61	male	Cancer
S06-1830	2	2	0	43	Male	Cancer
S06-1875	2	2	0	60	Male	Cancer

### RNA extraction and quality analysis

RNeasy FFPE kit was used for extraction of RNA from FFPE tissues according to manufacturer’s instructions. RNA was further purified using DNase I treatment (Ambion, Austin, TX) to eliminate any contaminating DNA. RNA concentrations were calculated using a Nanodrop ND-1000 spectrophotometer (NanoDrop, Wilmington, USA). RNA integrity was evaluated by running electropherograms and RNA integrity number, RIN (a correlative measure that indicates intactness of mRNA) was determined using the RNA 6000 PicoAssay for the Bioanalyzer 2100 (Agilent Technologies, Palo Alto, USA).

### Affymetrix miRNA arrays methods

The Affymetrix Genechip miRNA 4.0 array process was carried out according to the manufacturer’s instructions. 1000 ng RNA samples were labeled with the FlashTag™ Biotin using RNA Labeling Kit (Genisphere, Hatfield, PA, USA). The labeled RNA was further quantified, fractionated and hybridized to the miRNA microarray according to the standard protocol. The labeled RNA was then heated at 99 °C and then at 45 °C (for 5 mins at both temp). An Affymetrix® 450 Fluidics Station was used for RNA-array hybridization with agitation at 60 rotations per minute at 48 °C for 16 h. The chips were washed and stained using a Genechip Fluidics Station 450 (Affymetrix, Santa Clara, California, United States). The chips were then scanned with an Affymetrix GCS 3000 canner (Affymetrix, Santa Clara, California, United States). Using the Affymetrix® GeneChip™ Command Console software (AGCC) signal values were computed.

### Raw data preparation and statistic analysis

Raw data were extracted automatically in Affymetrix data extraction protocol using the software provided by AGCC. The CEL files import, miRNA level RMA + DABG-All analysis and result export using Affymetrix® Expression Console™ Software. Array data were filtered by probes annotated species. The comparative analysis between GC samples and control samples was carried out using independent *T*-test and fold-change where the null hypothesis was that no difference exists among two groups. False discovery rate (FDR) was further controlled by the adjustment of p value using Benjamini-Hochberg algorithm. All Statistical tests and visualization of differentially expressed genes were done using R statistical language v. 3.1.2.

### MiRNA quantification by real-time quantitative PCR (RT-qPCR)

For miRNA quantification SYBR Green RT-qPCR assay was used. Using miScript Reverse Transcription kit (Qiagen) one microgram of RNA was reverse-transcribed to cDNA. Further using miScript SYBR Green PCR kit (Qiagen) along with Universal primer and the miRNA-specific primers qPCR is performed in ABI 7900 Real-time PCR system (Applied Biosystems). The miRNA-specific primer sequences were designed using miRNA sequences obtained from the miRBase database. Each reaction mixture contained 10 μl of 2× Fast SYBR Green Master Mix (Life Technologies, Grand Island, NY), 0.5 μl of dye (ROX) II (50x), 1 μl of forward primer, 1 μl of reverse primer, 10 μL of distilled water, and 1 μl of cDNA template. Each sample was run in duplicates and U6 miRNA was used as control to normalize the expression levels of miRNAs. Mean expression values of each miRNAs (dublicate) relative to U6 RNA were calculated using the 2^-∆CT^ method [17], wherein ∆C_t_ = C_t(tumor)_-C_t(control)_ and control is the sample without treatment.

## Results and discussion

### Expression profiles of miRNA in gastric cancer

**Fig. 1:**
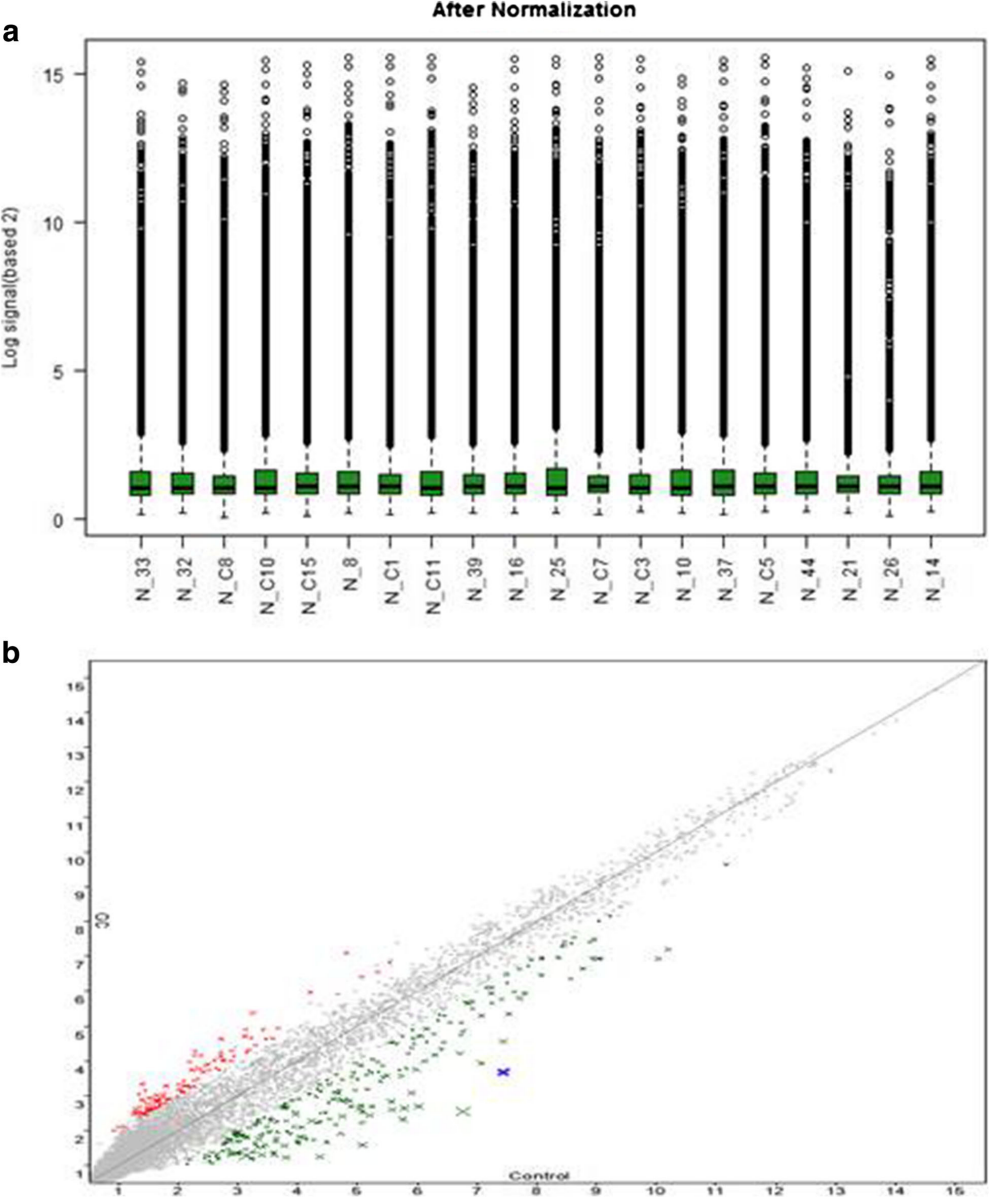
(**a**) Comparison of sample distribution using box plot (**b**) and scatter plot to show difference in miRNAs expression between cancerous and normal control samples

We used miRNA 4.0 array gene chips, to evaluate miRNA expression profiles between FFPE GC and normal tissues. By adjusting average change >2-fold and value <0.05 as a cut-off level, 1082 expressed genes were differentially expressed between the cancerous and normal control. Among them, one-way ANOVA showed that only 33 miRNAs had significant expression, where 8 (has-mir-193a-5p, has-mir-200c-3p, has-mir-1227-5p, has-mir-1909-3p, has-mir-1378, has-mir-3613-3p, has-mir-5196-5p and has-mir-7704) were apparently expressed in early stage cancer tissues (*p* < 0.05) and 25 (hsa-miR-22-3p, hsa-miR-28-3p, hsa-miR-29a-3p, hsa-miR-99a-5p, hsa-miR-27b-3p, hsa-miR-143-3p, hsa-miR-145-5p, hsa-miR-1251-5p, hsa-miR-126-3p, hsa-miR-185-5p, hsa-miR-200c-3p, hsa-miR-130b-3p, hsa-miR-378a-3p, hsa-miR-877-5p, hsa-miR-128, hsa-miR-378c, hsa-miR-3613-3p, hsa-miR-4532, hsa-miR-4668-5p, hsa-miR-4800-5p, hsa-miR-6124, hsa-miR-6812-5p, hsa-miR-7150, hsa-miR-7704, hsa-miR-7847-3p) were in advanced stage cancer samples as compare to normal control (*p* < 0.05) (Table. 2). As we used 2-fold as cut off value so some aberrant miRNAs in the chip analysis may be neglected. Differentially expressed genes were related to signal transduction, metabolism, angiogenesis, cell structure and cycle, and gene protein expression were mostly down-regulated. We found 13 new among 33 aberrant miRNAs that have not been reported before in GC studies. These miRNAs named as hsa-miR-1227-5p, hsa-miR-1909-3p, hsa-miR-3178, hsa-miR-7704 (in early-stage GC) and hsa-miR-1251-5p, hsa-miR-877-5p, hsa-mir-1281, hsa-miR-4532, hsa-miR-6124, hsa-miR-6812-5p, hsa-mir-7150, hsa-mir-7704 and hsa-mir-7847-3p (in late-stage GC) (Table. 2). There are many concerns about the integrity of miRNA from FFPE tissues and their suitability for microarray as RNA might be modified in chemical reaction and become fragmented in FFPE tissues [18] but due to very small size of miRNA (about 19-22 nucleotides) it’s not degraded in FFPE preparation. Many previous studies of miRNA microarray supporting a correlation in miRNA expression results from fresh and FFPE cancer tissues samples [19–21]. We found in all samples good value of RNA integrity and optical density (data not shown). In order to assess miRNA data after filtering we built box plot to visualize the distribution of data. The distributions of log2 ratios are almost same among miRNA chips. The probe-level data for the box plots are distributed from about 2–14 on the log scale (Fig. 1a). To check variation between cancer and normal tissues samples we assess the data using scatter-plot. Around 70–80 % miRNA seems to be same in cancerous and normal tissues used in this study (Fig. 1b).

**Table 2:** Expression profile of up and down-regulated miRNAs in GC patients

Transcript ID(Array Design)	Accession	Fold Change (linear) (GC vs. Control)	FDR *p*-value (GC vs. Control)
Detected in early stage (I and II)			
hsa-miR-193a-5p	MIMAT0004614	−2.06796	0.77052133
hsa-miR-200c-3p	MIMAT0000617	−22.552013	0.77052133
hsa-miR-1227-5p	MIMAT0022941	−1.717431	0.77052133
hsa-miR-1909-3p	MIMAT0007883	−1.951977	0.77052133
hsa-miR-3178	MIMAT0015055	−1.826664	0.72999652
hsa-miR-3613-3p	MIMAT0017991	2.981591	0.72999652
hsa-miR-5196-5p	MIMAT0021128	−1.959981	0.77052133
hsa-miR-7704	MIMAT0030019	−1.893422	0.72999652
U79	_	1.738033	0.74726754
Detected in early stage (III and IV)			
hsa-miR-22-3p	MIMAT0000077	−1.90434	0.10052633
hsa-miR-28-3p	MIMAT0004502	−1.87632	0.145678142
hsa-miR-29a-3p	MIMAT0000427	−1.96579	0.196109756
hsa-miR-99a-5p	MIMAT0000097	−1.97584	0.21331947
hsa-miR-27b-3p	MIMAT0000419	−2.29128	0.275381924
hsa-miR-143-3p	MIMAT0000435	−2.20679	0.132397944
hsa-miR-145-5p	MIMAT0000437	−2.20593	0.271934938
hsa-miR-1251-5p	MIMAT0028700	−2.70147	0.014814394
hsa-miR-126-3p	MIMAT0005903	−1.9555	0.292936143
hsa-miR-185-5p	MIMAT0001339	−1.55828	0.212836342
hsa-miR-200c-3p	MIMAT0000455	−2.18027	0.113424849
hsa-miR-130b-3p	MIMAT0000691	−2.41458	0.126769698
hsa-miR-378a-3p	MIMAT0000732	−1.78021	0.074619747
hsa-miR-877-5p	MIMAT0004949	1.696113	0.064059796
hsa-miR-1281	MIMAT0005939	−1.97312	0.064954696
hsa-miR-378c	MIMAT0016847	−2.51734	0.016444786
hsa-miR-3613-3p	MIMAT0017991	2.507303	0.212235198
hsa-miR-4532	MIMAT0019071	−2.05911	0.046570249
hsa-miR-4668-5p	MIMAT0019745	2.097354	0.268773791
hsa-miR-4800-5p	MIMAT0019978	1.510005	0.257957349
hsa-miR-6124	MIMAT0024597	2.156251	0.088004364
hsa-miR-6812-5p	MIMAT0027524	1.583428	0.250692071
hsa-miR-7150	MIMAT0028211	1.628161	0.277892706
hsa-miR-7704	MIMAT0030019	−1.64036	0.016444786
hsa-miR-7847-3p	MIMAT0030422	1.59577	0.244713443
U3	_	1.780994	0.060093686
U63	_	−1.69859	0.116804752
U3-2B	_	1.761599	0.070969781
U3-2	_	1.761599	0.070969781
U3-3	_	1.761599	0.070969781
U3-4	_	1.761599	0.070969781

### Identification of differentially expressed miRNAs in early vs late-stage GC

**Fig. 2:**
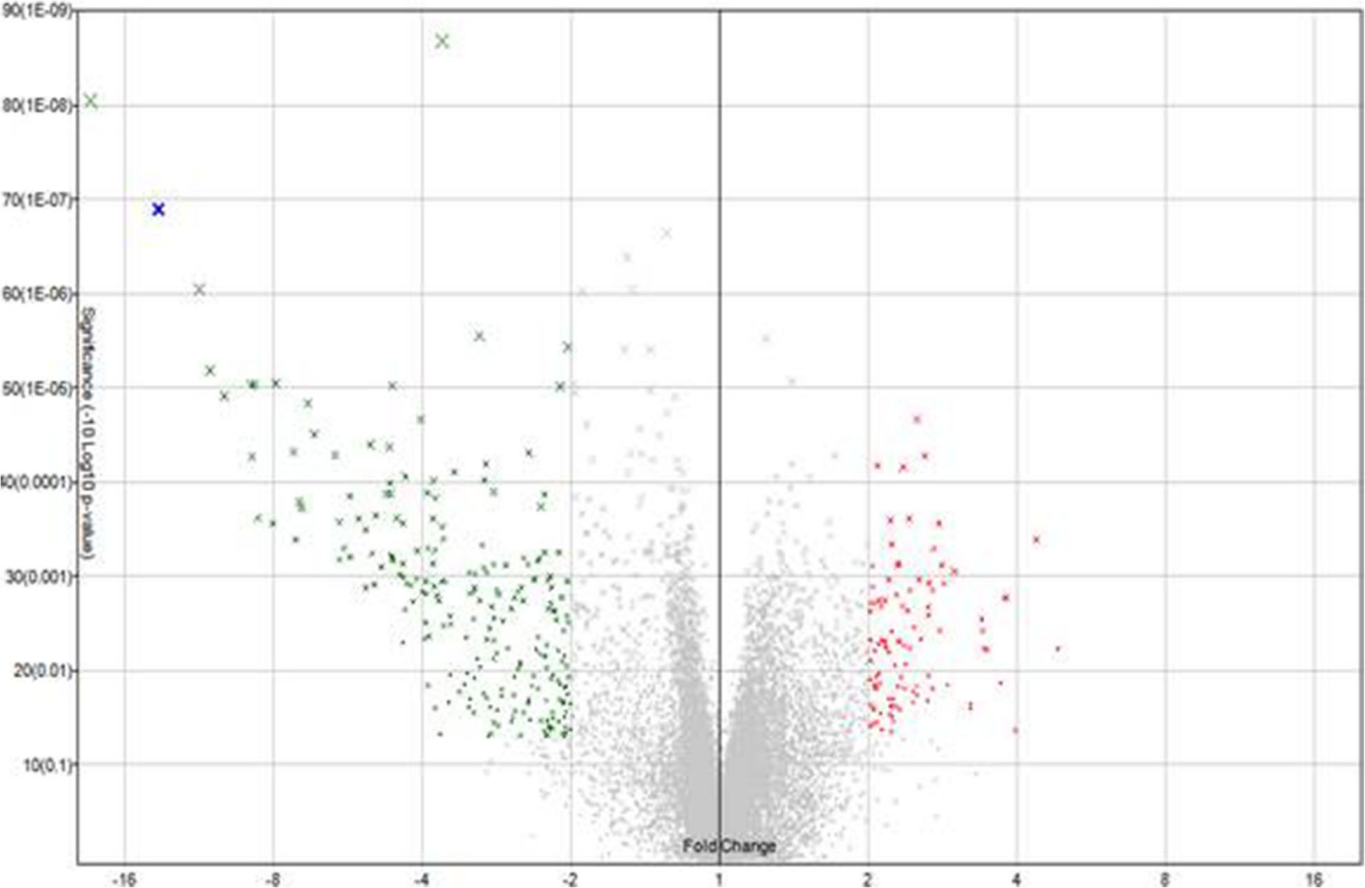
Volcano plot showing the relative expression of miRNA genes. The vertical lines correspond to 2.0-fold up and down, respectively, and the horizontal line represents a value of 0.05. The *red* point in the plot represents the differentially expressed genes with statistical significance

**Fig. 3:**
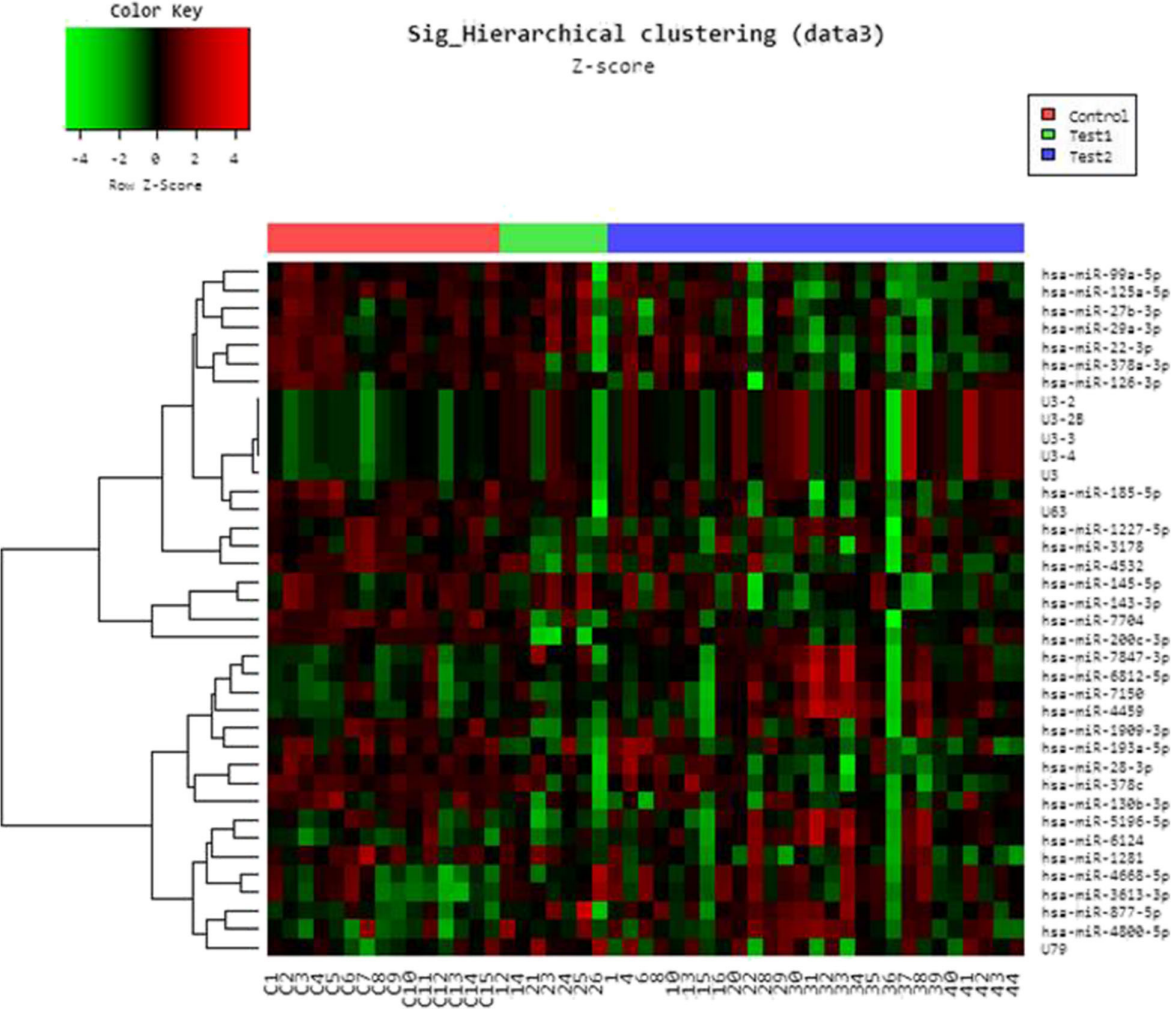
Hierarchical clustering for differentially expressed miRNAs in cancer versus normal pass volcano plot. *Red* indicates high relative expression and *green* indicates low relative expression

We analyzed the expression of miRNAs significantly expressed from cancerous (early and last stage) and normal tissues using Volcano plot filtering. For up and down-regulated miRNAs genes we used ≥ 2.0 fold change and value *P* value (<0.05). In this plot, differentially expressed genes are statistically significant and are shown in red and green points (Fig. 2). We identified 1082 differently expressed miRNA and only 33 had significant expression, where 9 genes showed up-regulation and 24 genes showed down-regulation of expression in GC (early and late-stage GC) as compare to normal gastric tissues. Further hierarchical clustering analyses (Fig. 3) were performed on the basis of differentially expressed miRNAs from cancerous versus normal tissues. Based on this hierarchical clustering, we have divided miRNAs expression in three groups: early-stage, late-stage and normal tissues. These results showed change in cancerous and control tissue samples. We have identified many new miRNAs from this study from Saudi population that have never been reported in studies related to cancer as well as in GC. Among 33 aberrant miRNAs (early and late-stage GC) 13 were new and rest of the miRNAs were reported before in cancer studies. Similarly, from remaining 20 miRNAs, only 9 were reported in GC studies before. Most of the miRNAs were down-regulated only 9 were up-regulated. In early-stage GC tissue samples, 8 miRNAs had aberrant expression as compare to normal control. Among them 7 was up-regulated and only 1 showed down-regulation (Table. 2). Some of them were not reported before in GC except has-mir-200c-3p. Some miRNAs identified in early-stage cancer tissues samples, including hsa-miR-193a-5p, hsa-miR-3613 and hsa-miR-5196-5p were reported already in cervical cancer and acute lymphoblastic leukemia [22, 23]. In our study, significant down-regulation of has-mir-200c-3p has been seen in early and late-stage GC tissue samples.

The miR-200 family consists of five members and is encoded by two genes on chromosomes 12 and function as tumor regulator [24, 25]. It plays an important role in different types of cancer by inhibition, invasion, migration, proliferation and drug resistance. Previous studies have shown down-regulation of miR-200c in GC that is consistent with our study [26, 27]. The miR-200c has been reported in various types of cancers, including colorectal [28], breast [29], cholangiocarcinoma [30], colorectal [31], hepatocellular [32], lung adenocarcinoma [33], ovarian [34] and renal cell carcinoma [35]. The miR-200 family inhibits the epithelial-mesenchymal transition and metastasis by down-regulating *ZEB1* and *ZEB2* (Zinc-finger E-box Binding homeobox 1 and 2). The miR-200 inhibits angiogenesis by regulating interleukin-8 and CXCL1 secreted by cancer cells. Marked reduction of angiogenesis has been observed after delivering miR-200 members into the tumour endothelium [36]. Another study reported that extracellular matrix proteins and peptidases are targeted by miR-200 and alters the tumor microenvironment to inhibit metastasis [37]. *H. pylori* induced infection is another risk factor for progression of GC. Matsushima et al. [38] have characterized that decreased expression of tumor suppressor family miR-200 has been seen in *H.pylori* positive GC patients. Few other studies reported down-regulation of miR-200 in *H. pylori*-infected GC and increased expression of anti-apoptotic proteins, Bcl-2 and XIAP hence inhibiting apoptosis [39–41].

In late-stage GC tissue samples, 8 miRNAs were up-regulated and 17 showed down-regulation with 10 new miRNAs (has-mir-1251-5p, has-mir-877-5p, has-mir-1281, has-mir-378c, has-mir-4532, has-mir-6124, has-mir-6812-5p, has-mir-7150, has-mir-7704 and has-mir-7847-3p) not reported before (Table. 2). Many miRNAs involve in tumorigenesis and altered in variety of cancers are detected in our study such as tumor-suppressor (has-mir-22-3p, has-mir-28-3p, has-mir-99a-5p, has-mir-130b-3p, has-mir-378a-3p, has-mir-3613-3p, has-mir-4668-5p and has-mir-4800-5p) are reported before in colorectal [42], cervical [22] and breast cancer [43]. Some of the miRNAs (has-mir-29a-3p, has-mir-27b-3p, has-mir-143-3p, has-mir-145-5p, has-mir-126-3p, has-mir-185-5p, has-mir-200c-3p) differential expressed in late-stage GC samples had similar kind of expression in previously published GC studies. The down-regulation of has-miR-29a-3p in our study is consistent with previous study of GC where decreased expression of has-miR-29a-3p promotes cell proliferation by suppressing the expression of cell cycles regulators [44, 45]. The receptor tyrosine kinase like orphan receptor 1 (ROR1) protein is an oncogenic protein. The miR-27b-3p suppress the expression of ROR1 in GC hence function as tumor suppressor [46]. Similar expression of has-mir-27b-3p has been detected in our study. Furthermore, other GC related miRNAs such as has-mir-143-3p, has-mir-145-5p, has-mir-126-3p, has-mir-185-5p have found to be down-regulated in our study have shown similar expression in other studies and reported as tumor-suppressors [47–49]. In addition to miRNAs, we found seven small nucleolar RNA (snoRNAs) differentially expressed in both early and late-stage tumor samples. In our study, only 1 snoRNA (U63) showed down-regulation and others (U79, U3, U3-2b, U3-2, U3-3 and U3-4) were up-regulated and are related to human C/D box group (Table. 2). Several recent studies have highlighted the role of snoRNAs as miRNAs precursors and have similar function like miRNAs [50, 51]. Another study on Epstein-Barr virus genome, suggests their role in infection [52]. What role these snoRNAs may play in GC is interesting to explore.

The present study has limitations due to small number of tumor samples (*n* = 34), especially samples of early stages. Therefore, results of the present study need further validation using larger group of GC patients in future. Our results showed that significant down-regulation of has-mir-200c-3p is markedly observed in early-stage GC as compare to late-stage GC samples.

### Validation of dysregulated miRNAs by quantitative PCR analysis

**Fig. 4:**
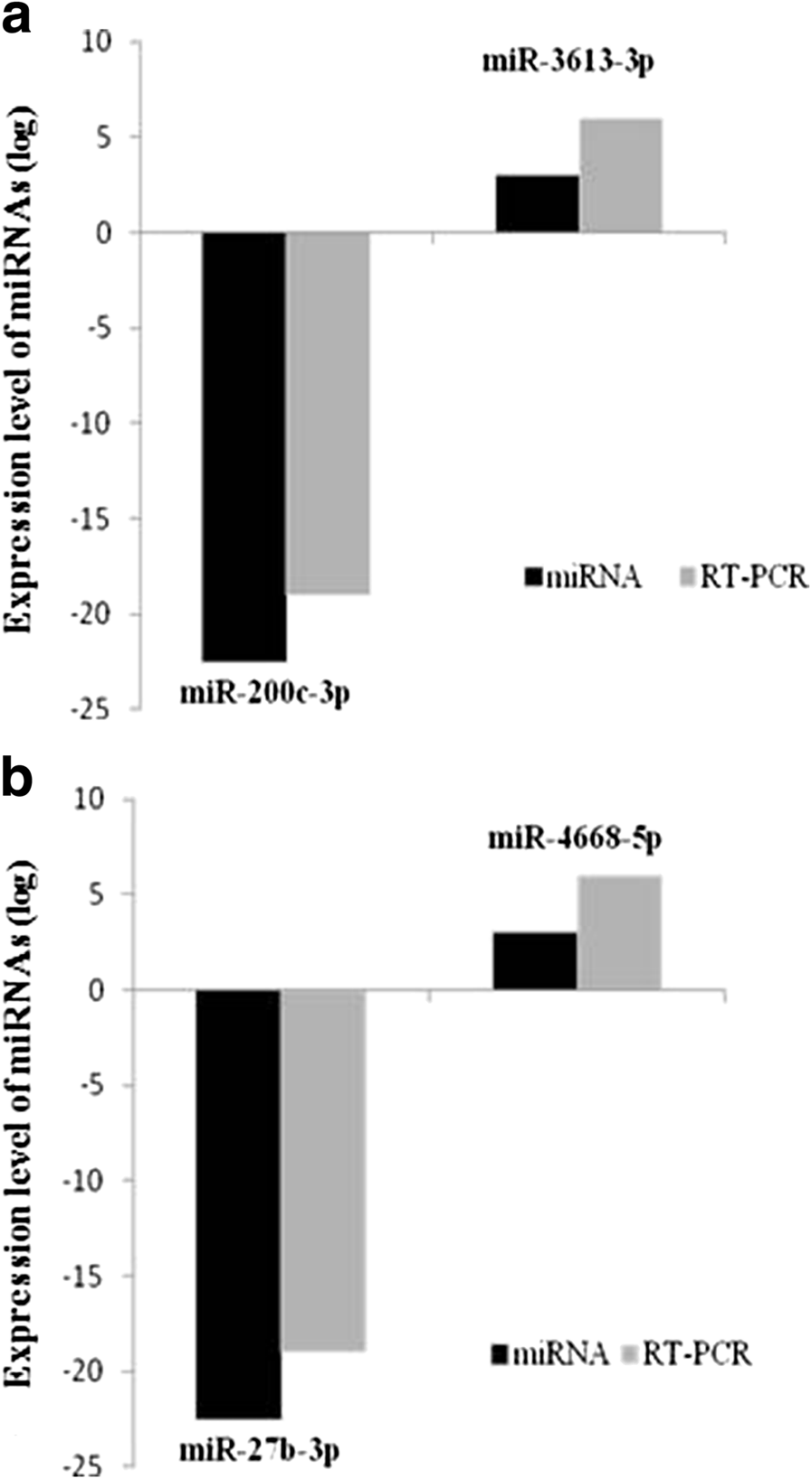
Quantification of the highly expressed miRNAs in the human GC versus normal control in four selected upregulated miRNA. (**a**) Early-stage and (**b**) Late-stage miRNAs

Further using real-time quantitative PCR analysis, results from expression array were validated. We selected four significantly aberrant miRNAs with 2-fold change including hsa-miR-200c-3p, hsa-miR-3613, hsa-miR-27b-3p, hsa-miR-3613 from both stages to test in cancerous versus normal tissues and miRNA U6 was used as control. The miRNA expression analysis of 2 oncogenic hsa-miR-200c and hsa-miR-27b-3p was significantly down-regulated while hsa-miR-3613 and hsa-miR-3613 showed up-regulation in comparison with normal control (Fig. 4a and b) that is consistent with miRNAs array results. Significantly down-regulation of hsa-miR-200c has been reported previously in many studies of cancer indicating the role of hsa-miR-200c as tumor suppressor [24–34]. hsa-miR-200c had multiple roles in regulating tumor cell growth by inhibiting the metastasis and invasion of hepatocellular carcinoma and gastric carcinoma [35, 36]. At last, accordant down-regulation of hsa-miR-200c in GC tissues was detected, in comparison with the normal tissues. We also found aberrant expression of some new miRNAs in GC patients, which have no report of aberrant expression in any other studies.

## Conclusions

In conclusion, we explored the miRNAs expression of FFPE gastric tissues from GC patients and normal control. Many miRNAs showed aberrant expression in cancerous versus normal control. As it is evident from many previous studies and also the current results strongly suggest that hsa-miR-200c acts as a tumor suppressor miRNA that plays a potential role in the oncogenesis in humans. Hence highlighting its functions as a tumor-suppressive miRNA and prognostic marker in GC patients in Saudi population. The significance and role of aberrant miRNAs expression of GC patients in Saudi population will be better understood as more miRNAs will be identified. Our study may be helpful in future to identify potential prognostic biomarkers for GC.

## Abbreviations

GC: Gastric cancer
miRNAs: microRNAs
FFPE: Formalin-fixed paraffin-embedded
qPCR: Real-time quantitative PCR
